# Frequency-Following Response (FFR) in cochlear implant users: a systematic review of acquisition parameters, analysis, and outcomes

**DOI:** 10.1590/2317-1782/20212021116

**Published:** 2022-01-19

**Authors:** Leonardo Gleygson Angelo Venâncio, Mariana de Carvalho Leal, Laís Cristine Delgado da Hora, Silvana Maria Sobral Griz, Lilian Ferreira Muniz

**Affiliations:** 1 Programa de Pós-graduação em Saúde da Comunicação Humana, Universidade Federal de Pernambuco – UFPE - Recife (PE), Brasil.; 2 Programa de Pós-graduação em Saúde da Comunicação Humana, Departamento de Cirurgia, Universidade Federal de Pernambuco – UFPE - Recife (PE), Brasil.; 3 Programa de Pós-graduação em Saúde da Comunicação Humana, Departamento de Fonoaudiologia, Universidade Federal de Pernambuco – UFPE - Recife (PE), Brasil.

**Keywords:** Cochlear Implant, Evoked Potentials Auditory Brain Stem, Speech, Auditory Perceptual Disorders, Electrophysiology, Review

## Abstract

**Purpose:**

To characterize the acquisition parameters, analysis, and results of the frequency-following response (FFR) in cochlear implant users.

**Research strategies:**

The search was conducted in Cochrane Library, Latin American and Caribbean Health Sciences Literature (LILACS), Ovid Technologies, PubMed, SciELO, ScienceDirect, Scopus, Web of Science, and gray literature.

**Selection criteria:**

Studies on FFR in cochlear implant users or that compared them with normal-hearing people, with no restriction of age, were included. Secondary and experimental studies were excluded. There was no restriction of language or year of publication.

**Data analysis:**

The data were analyzed and reported according to the stages in the Preferred Reporting Items for Systematic Reviews and Meta-Analyses (PRISMA), 2020. The methodological quality was analyzed with the Joanna Briggs Institute Critical Appraisal Checklist for Analytical Cross-Sectional Studies. Divergences were solved by a third researcher.

**Results:**

Six studies met the inclusion criteria. Only one study was comparative, whose control group comprised normal-hearing people. The variations in acquisition parameters were common and the analysis predominantly approached the time domain. Cochlear implant users had different FFR results from those of normal-hearing people, considering the existing literature. Most articles had low methodological quality.

**Conclusion:**

There is no standardized FFR acquisition and analysis protocol for cochlear implant users. The results have a high risk of bias.

## INTRODUCTION

The evolution of cochlear implant (CI) technology has enabled speech perception for its users’ oral acquisition, making it an effective resource in hearing loss (HL) rehabilitation^(1)^. However, variations in CI users’ auditory and linguistic performance have been frequently reported^(2)^ in comparison with normal-hearing people. This suggests they possibly have different verbal auditory processing.

Although the precise origin of such variability has not been fully established, individual characteristics, HL factors, and CI characteristics are believed to influence IC users’ speech perception^(3)^. Behavioral tests are traditionally used to assess verbal auditory processing. However, as they require the patient’s cooperation, their application tends to be limited in new CI users and nonverbal children^(4)^.

Therefore, objective tests are an alternative in these cases. One of them is the frequency-following response (FFR), a noninvasive procedure that measures speech decoding with the neurons’ synchronous activity in the auditory pathways – especially in the brainstem, an important region to the language and auditory functions^(5)^.

Increasing evidence shows that the FFR aids the differential diagnosis of language and auditory processing disorders^(6-8)^. Hence, the examination must be properly recorded, which depends on the acquisition and analysis parameters being used^(9)^. Studies with FFR in CI users apply similar parameters to those in normal-hearing people, with no standardization previous to the examination^(10-12)^.

This is a challenging scenario because choosing inadequate parameters may contaminate the FFR records. In this regard, the electrical artifact generated by the CI makes the clinical interpretation of the results more difficult, setting an inherent limitation to its possible applicability in CI users^(13)^.

Various techniques to minimize CI artifacts have been described in the literature. However, they were applied to the cortical potentials and/or auditory steady-state response^(14,15)^. No standardized protocol to obtain FFR free from CI artifacts is known. Neither is it clear whether there are valid differences in response patterns between CI users and normal-hearing people, which makes it difficult to identify inconsistencies and fragilities in the evidence available in order to minimize them in the future.

## OBJECTIVE

To characterize the acquisition and analysis parameters and the results in FFR examination in CI users with a systematic review of the literature.

## RESEARCH STRATEGY

The systematic review of the literature was written following the Preferred Reporting Items for Systematic Reviews and Meta-Analyses (PRISMA) 2020^(16)^ and the Meta-analysis of Observational Studies in Epidemiology (MOOSE)^(17)^. The complete research protocol is published under registry CRD42020151073 in the International Prospective Register of Systematic Reviews (PROSPERO).

The bibliographic survey was conducted in Cochrane Library, Latin American and Caribbean Health Sciences Literature (LILACS), Ovid Technologies, PubMed, SciELO, ScienceDirect, Scopus, and Web of Science. The gray literature was searched in British Library Inside, DissOnline.de, OAIster, openDOAR, OpenGrey, and The New York Academy of Medicine (NYAM). The searches took place on March 10, 2020, and were updated on October 10, 2020.

The search strategy included descriptors and keywords that describe the population and examination, namely: “Cochlear Implants”; “Cochlear Implantation”; “Evoked Potentials, Auditory, Brain Stem”; *“Implante Coclear”; “Potenciais Evocados Auditivos do Tronco Encefálico”; “Implantação Coclear”; “Implantes Cocleares”; “Respostas Evocadas Auditivas do Tronco Encefálico”*; “Frequency-Following Response”; “Auditory brainstem response to complex sounds”; “envelope-following response”; “speech-evoked auditory brainstem response”; “Speech-evoked ABR”; subcortical steady-state response.

To ensure the retrieval of as many relevant studies as possible, no comparison group and outcome limiters were used. The terms were combined and crossed with the Boolean operators (OR and AND), and, when applicable, orthographic and syntactic variations and synonyms were used to broaden the scope and ensure considerable precision.

## SELECTION CRITERIA

Studies that examined CI users and that compared them with normal-hearing people were selected, as long as they described at least one of the following acquisition parameters: transducer, speech stimulus characteristics (type, duration, intensity, and polarity), ear, electrode fixation, stimulus presentation rate, reproducibility, filters, impedance, and artifact rejection. No restriction was used regarding the year and language of publication.

Studies whose subjects had neurological or genetic syndromes, brainstem malformations, language or hearing disorders, or took medications that act on the central nervous system, as well as in vitro and ex vivo experiments with animals, reviews, editorials, indices, news, notes, letters, abbreviations, appendices, reports, books, and book chapters were excluded.

## DATA ANALYSIS

The authors (L.G.A.V. and L.C.D.H.) independently carried out the data analysis in four stages (identification, screening, eligibility, and inclusion)^(16)^. If they did not agree in the inclusion stage, the study was evaluated by a third researcher (L.F.M) to make the final decision.

In the identification stage, appropriate studies were selected through a database search. The Mendeley Desktop reference manager (version 1.19.8)^(18)^ was used to manage, store, and share the studies and to remove the duplicates. In the screening stage, the titles and abstracts were read to dismiss studies that did not meet the preestablished selection criteria and maintain possibly eligible studies.

In the eligibility stage, after reading the full texts, we decided which studies corresponded to the review approach: FFR acquisition in CI users or the comparison between CI users and normal-hearing people. In the inclusion stage, the studies that met the criteria in all the previous stages formed the sample for data extraction.

The collected data included details of the studies (author, year, and place), study design, sample characteristics (population, sample size, and age), potential confounding variables (on the HL and CI characteristics), acquisition protocol, FFR analysis, and results. This information was entered into a Microsoft Office Excel^®^ spreadsheet.

The methodological quality of the studies was analyzed with the Joanna Briggs Institute (JBI) Critical Appraisal Checklist for Analytical Cross-Sectional Studies^(19)^.

## RESULTS

### Study selection and characteristics

A total of 6,639 articles were identified – 97.60% (N = 6.480) of them through the systematic search in the databases and 2.39% (N = 159) in the gray literature. After removing the duplicates, 5,245 articles were screened. In the title and abstract screening, 5,238 articles were excluded because they did not meet the eligibility criteria.

**Figure 1:**
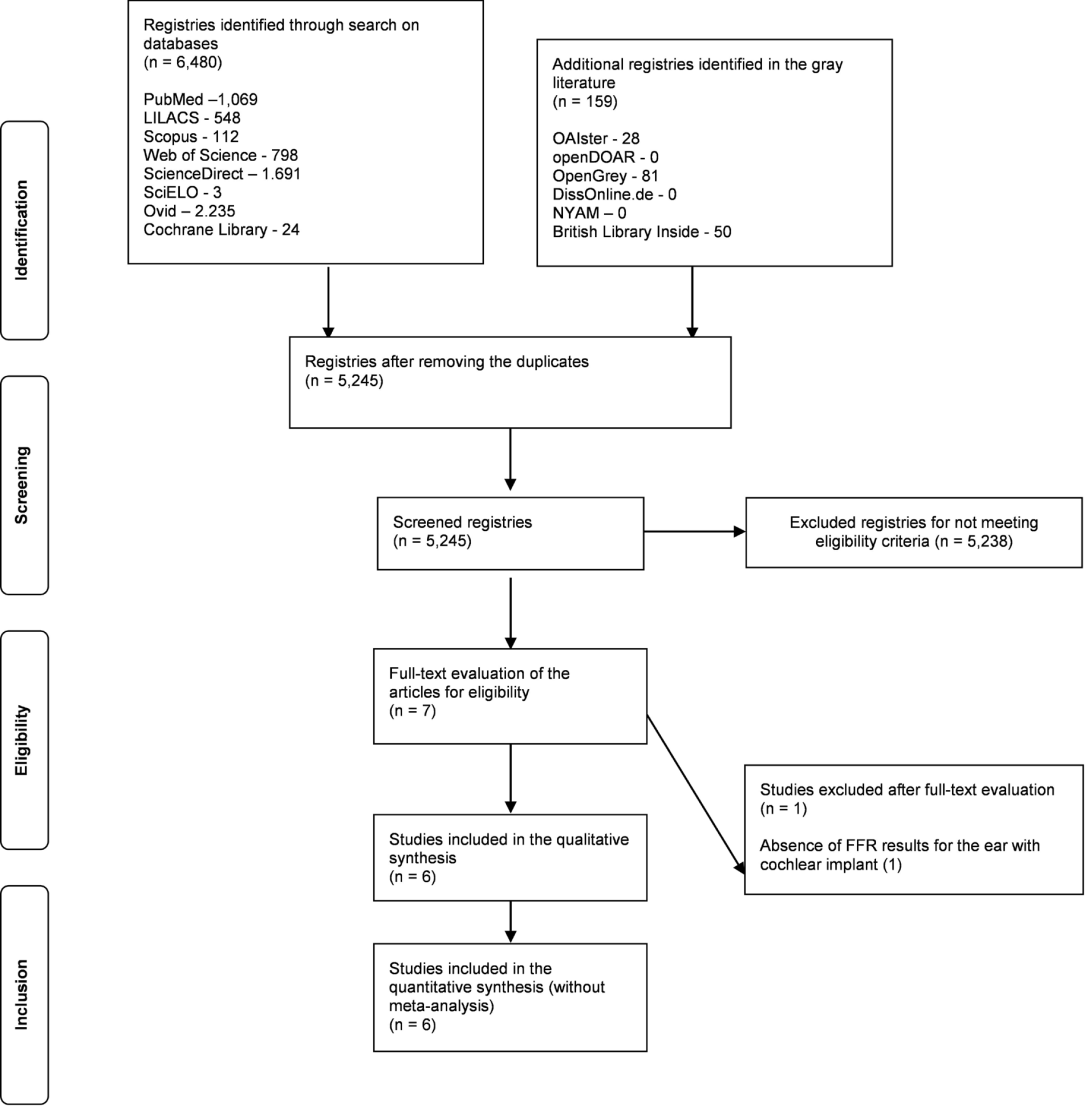
Flowchart with the study selection process

After screening the titles and abstracts, seven articles remained for full-text reading. In the eligibility stage, one study^(20)^ was excluded because it lacked data on the implanted ear. Hence, only six studies were included^(10–13,21,22)^ for data extraction (Figure 1).

The characteristics of the six studies included in the review are presented in Table 1. The studies were carried out in 2015^(10)^, 2016^(11)^, 2017^(12)^, 2018^(21)^, and 2020^(22)^. Four studies were from Egypt^(10-12,21)^, one from the United Kingdom^(13)^, and one from Iran^(22)^. All studies were observational. Only one study^(22)^ had a comparison group, with a population of normal-hearing children. The sample distribution varied in subcategorized groups that followed the originating issues in each primary research.

**Table 1:** Characteristics of the studies, analysis method, results, and possible effect of the categorization variables in the frequency-following response in cochlear implant users

	Author, year, and place										
	BinKhamis et al, 2019; United Kingdom^(13)^	Gabr et al, 2015; Egypt^(10)^		Gabr et al, 2018; Egypt^(21)^		Jarollahi et al, 2020; Iran (22)		Mourad et al, 2016; Egypt^(11)^	Rahman et al,2017; Egypt^(12)^		
Variables											
Population and sample size (N)											
Group 1 (G1)	Cochlear implant users (12)	Good cortical records in speech-evoked cortical auditory potentials (9)		Language improvement quotient > 0.7 (20)		Cochlear implant users (20)		Cochlear implant users (10)	1 year using the cochlear implant (10)		
Group 2 (G2)	-	Poor cortical records in speech-evoked cortical auditory potentials (11)		Language improvement quotient < 0.7 (20)		Normal-hearing people (20)		-	2 years using the cochlear implant (12)		
Group 3 (G3)	-	-		-		-		-	3 years using the cochlear implant (9)		
Age (Years old)											
	39-60	2-6		4-10		8-10		5-1-	4-5		
Hearing loss (n)											
Onset of Hearing loss	Postlingual (12)	Pre- and postlingual (20)		Prelingual (40)		Prelingual (20)		Prelingual (10)	Prelingual (31)		
Degree of Hearing loss	NR	Severe to profound (20)		Severe to profound (40)		Profound (20)		NR	NR		
Etiology of Hearing loss	Mumps (1), Idiopathic (6), Meningitis (2), rubella (2), trauma (1)	Hereditary (10), post-febrile (2), idiopathic (8)		NR		NR		NR	Hereditary and idiopathic (not specified)		
Cochlear implant (n)											
Right ear	6	9		NR		20		10	25		
Age in years when began to use	NR	2.2 for G1 and 3.2 for G2		4.1 for G1 and 3.8 for G2		4.1 for G1		NR	2.5 for G1, 2,1 for G2 and 1,5 for G3		
Mean time of use in years (group)	7.2	NR		2.6 for G1 and 2.4 for G2		4.6		NR	1.0 for G1, 2.0 for G2 e 3.0 for G3		
Manufacturer (n)											
Abionic	-	5		NR		-			-		
Cochlear	6	5		-		20		-	-		
Med-El	6	10		-		-		10	22		
Neurelec	-	-		-		-		-	9		
Speech processing strategy											
ACE (MP1+2)	4	NS		NS		20		-	NS		
FS4	6	-		-		-		10	-		
SPEAK (BP+1/BP+2)	2	-		-		-		-	-		
FFR analysis method											
Peak latency	Yes	Yes		Not		Yes		Yes	Yes		
Amplitude	Yes	Yes		Not		Yes		Yes	Yes		
Duration of the VA complex	Not	Not		Yes		Not		Not	Not		
Slope of the VA complex	Not	Not		Yes		Not		Not	Not		
Area of the VA complex	Not	Not		Yes		Not		Not	Not		
Amplitude of the VA complex	Not	Not		Yes		Not		Not	Not		
Spectral magnitudes	Not	Not		Not		Yes		Not	Not		
FFR results per groups											
	G1	G1	G2	G1	G2	G1	G2	G1	G1	G2	G3
Latency values (ms)											
V	8.22	9.60	10.3	-	-	11.9	9.51	2.59	9.35	8.65	7.27
A	12.1	12.9	14.7	-	-	13.4	10.9	4.5	10.6	9.86	9.20
C	NR	23.4	26.9	-	-	24.3	20.4	19.7	14.0	13.0	15.3
D	22.9	31.0	35.1	-	-	27.7	25.2	23.3	20.5	20.3	22.2
E	29.7	38.7	45.0	-	-	36.5	34.5	31.6	26.6	25.1	24.1
F	38.3	48.0	53.3	-	-	45.6	42.9	NR	38.0	39.0	39.8
O	46.2	56.9	63.0	-	-	54.1	51.1	NR	46.3	52.0	45.7
Amplitude values (µV)											
V	NR	NR	NR	-	-	0.12	0.17	14.7	NR	NR	NR
A	0.96	NR	NR	-	-	-0.14	-0.20	9.74	NR	NR	NR
C	NR	NR	NR	-	-	-0.09	-0.14	2.11	NR	NR	NR
D	2.04	NR	NR	-	-	-0.26	-0.18	6.29	NR	NR	NR
E	0.79	NR	NR	-	-	-0.24	-0.17	7.27	NR	NR	NR
F	1.49	NR	NR	-	-	-0.18	-0.12	NR	NR	NR	NR
O	0.87	NR	NR	-	-	-0.12	-0.16	NR	NR	NR	NR
Slope of the VA complex (µV/ms)	-	-	-	1.88	0.47	-	-	-	-	-	-
Area of the VA complex (µV X ms)	-	-	-	18.6	9.55	-	-	-	-	-	-
Amplitude of the VA complex (µV)	-	-	-	5.06	2.11	-	-	-	-	-	-
Duration of the VA complex (ms)	-	-	-	3.70	3.22	-	-	-	-	-	-
Spectral magnitude measure values (µV)											
HF	-	-	-	-		7.04	9.89	-	-	-	-
F0	-	-	-	-		2.25	4.58	-	-	-	-
F1	-	-	-	-		0.44	1.68	-	-	-	-
Possible effect of the categorization variables in FFR	The Cochlear implant model Nucleus 22 with the SPEAK strategy resulted in responses similar to the speech envelope, with smaller amplitude before removing artifacts	NR		The age when they began to use the Cochlear implant had a positive correlation with the VA complex amplitude, area and slope measures		NR		NR	Me time of Cochlear implant use in years had a positive correlation with the latencies of waves V and E and a negative correlation with the latency of wave O		

**Caption:** ACE – Advanced Combination Encoder; BP – bipolar stimulation; F0 – fundamental frequency; F1 – first formant; FS4 – fine structure four; HF – high frequencies; CI – cochlear implant; LIQ – language improvement quotient; MP – monopolar stimulation; NS – not specified; NR – not reported; RMS – effective value; S-CAEPs – speech-evoked cortical auditory potentials; SPEAK – Spectral Peak

The sample size ranged from 10^(11)^ to 40^(21)^ CI users, and their age ranged from 2^(10)^ to 60 years^(13)^. Five studies were conducted with children^(10-12,21,22)^.

All studies had a high risk of bias due to the low methodological quality, which is ascribable to limitations in inclusion criteria, description of participants, setting of the study, and identification of the confounding factors (Table 2).

**Table 2:** Classification of the methodological quality of the studies according to the criteria of the Joanna Briggs Institute (JBI) Critical Appraisal Checklist for Analytical Cross-Sectional Studies

	BinKhamis et al., 2019^(13)^	Gabr et al., 2015^(10)^	Gabr et al., 2018^(21)^	Jarollahi et al., 2020^(22)^	Mourad et al., 2016^(11)^	Rahman et al., 2017^(12)^
1. Were the criteria for inclusion in the sample clearly defined?	N	Y	Y	Y	N	Y
2. Were the study subjects and the setting described in detail?	N	N	N	Y	N	N
3. Was the exposure measured in a valid and reliable way?	N	N	N	Y	N	Y
4. Were objective, standard criteria used for measurement of the condition?	N	Y	N	Y	N	Y
5. Were confounding factors identified?	N	N	N	N	N	N
6. Were strategies to deal with confounding factors stated?	N	N	N	N	N	N
7. Were the outcomes measured in a valid and reliable way?	Y	Y	Y	Y	Y	Y
8. Was appropriate statistical analysis used?	N	Y	Y	Y	N	Y
Adequate/Total	1/8	4/8	3/8	6/8	1/8	5/8

**Caption:** Y – Yes; N – No; U – Unclear; NA – Not applicable

### Acquisition parameters and FFR analysis in CI users

The FFR acquisition parameters most used in the studies with CI users are shown in Table 3. There were frequent variations in parameters, except for the transducer, type of stimulus, mode of stimulation, and polarity of the stimulus. All examinations were performed with loudspeakers, speech stimulus /da/ with alternating polarity, through monoaural stimulation in the CI ear.

**Table 3:** Frequency-following response acquisition parameters in cochlear implant users in the studies included in the review

**Study**	**Positioning of the loudspeaker**	**Characteristics of the speech stimulus /da/**			**Ear**	Positioning of the electrodes*	**Presentation rate (stim/ms)**	**Total number of sweepings**	**Window (ms)**	**Filters (high-pass – low-pass) (Hz)**	**Impedance of the electrodes / artifact rejection**	**Strategy to diminish the CI artifact**
		Duration (ms)	Intensity	Polarity								
BinKhamis et al., 2019^(13)^	45° azimuth, 1.1 meters away from the CI speech processor microphone	40	70 dB-A	Alternating	RE/LE	Cz (+), A1 and A2 (-), and Fz (neutral)	9.1	10,000	NR	100 – 3000	< 3 kΩ / NR	YES
Gabr et al., 2015^(10)^	45° azimuth, 50 cm away from the ear with CI	206	70 dBHL	Alternating	RE/LE	Fz (+), M1 and M2 (-), and Fpz (neutral)	11.1	3,072	75	150 – 1500	NR / NR	NR
Gabr et al., 2018^(21)^	45° azimuth, 1 meter away from the ear with CI	NR	70 dBHL	Alternating	NR	Fz (+), M1 and M2 (-), and Fpz (neutral)	11.1	NR	75	150 – 1500	NR / NR	NR
Jarollahi et al., 2020^(22)^	45° azimuth, 1 meter away from the ear with CI	40	50 dBSL	Alternating	RE	Cz (+), A1 and A2 (-), and Fpz (neutral)	9.1	4,000	60	100 – 2000	< 5 kΩ / NR	NR
Mourad et al., 2016^(11)^	90° azimuth, 30 cm away from the head	40	70 dBHL	Alternating	RE	Fpz, M1, and chin (neutral)	2.1	1,000	60	30 – 3000	< 3kΩ / NR	NR
Rahman et al., 2017^(12)^	0° azimuth, 1 meter away from the participant	40	80 dBSPL	Alternating	RE/LE	Cz (+), M1 (-), and FPz (neutral)	10.9	6,000	62	100 – 2000	< 5 kΩ / ±31 mV (myogenic)	YES

**Caption:** (*) – based on the International 10-20 System (A1 = left earlobe; A2 = right earlobe; Cz = vertex; Fpz = forehead; Fz = front; M1 = left mastoid; M2 = right mastoid); (-) – reference electrode; (+) – active electrode; dB – decibel; stim – stimuli; NR – not reported; RE – right ear; LE – left ear; SPL – sound pressure level

The loudspeaker was positioned at 45° azimuth in four studies^(10,13,21,22)^. However, the distance from the participant to the loudspeaker ranged from 50 cm^(10)^ to 1.1 meters^(13)^. Regarding speech stimulus characteristics, four studies used the syllable /da/ lasting 40 ms^(11-13,22)^, and three studies presented the speech stimulus at 70 dBHL^(10,11,21)^.

The FFR was recorded with positive, reference, and ground electrodes, respectively fixed, in two studies, on the upper front (Fz), mastoids (M1 and M2), and forehead (Fpz)^(10,21)^. Two other studies used another configuration possibility^(13,22)^: vertex (Cz), earlobes (A1 and A2), and forehead (Fpz). The most frequently used stimulus presentation rates were 9.1^(13,22)^ and 11.1^(10,21)^. The total number of sweepings ranged from 1,000^(11)^ to 10,000 stimuli^(13)^. The most frequent examination visualization window lasted 60 ms^(11,22)^ and 75 ms^(10,21)^.

Two studies used 100 Hz high-pass and 2000 Hz low-pass filters^(12,22)^. The electrode impedance was kept below 3 (kΩ)^(11,13)^ and below 5 kΩ^(12,22)^ in two studies. Only one study^(12)^ used artifact rejection to minimize myogenic interferences. Two studies tried to diminish CI artifact with a single-channel acquisition approach^(13)^ and electrode jumper^(12)^.

The peak latency and amplitude were the predominant analysis measures in the six studies included in the review. The studies used the absolute mean latency and amplitude values of waves V, A, C, D, E, F, and O, as well as the duration, slope, and area of the VA complex (Table 1).

Only one study^(22)^ used the Fourier analysis to examine the frequency domain representation with spectral measures of fundamental frequency (F0), first formant (F1), and high frequencies (HF). On the other hand, two studies^(11,22)^ analyzed the amplitude effective value (RMS) and the cross-correlation between the stimulus and the FFR.

### FFR results in CI users

The FFR results in the studies are shown in Table 1. In general, CI users had delayed latencies, smaller amplitudes, higher VA complex slope, area, duration, and amplitude values, and lower spectral measures (HF, F0, and F1) than the data established in the literature for normal-hearing children^(8,23)^ and adults^(7,24)^.

The studies had clinical and methodological diversities, limited samples available for comparison, and categorized subgroups of the same population (CI users), which make the findings heterogeneous. Hence, it was not possible to make the meta-analysis and the certainty of evidence for the lack of sufficient data to calculate the effects sizes.

In summary, this systematic review of the literature approaches a current perspective on the possible application of FFR in CI users. The FFR has proved to be a reliable examination to investigate the integrity of speech processing in different populations^(6–8)^. Nevertheless, in CI users this examination tends to be limited, with various caveats in result interpretation.

In the following sessions, the FFR acquisition and analysis parameters in CI users, the results found, and the possible effects of the confounding variables related to the examination are discussed and analyzed for better comprehension.

### Analysis of the FFR acquisition and analysis parameters in CI users

Variations in the FFR acquisition parameters affect the examination recordings^(5,25)^. Since all studies presented the stimuli with loudspeakers, and none of them reported a previous calibration, a temporal compensation was expected due to the distance from the transducer to the CI and the time the CI processor takes to process the acoustic signals^(26)^. Moreover, the authors failed to elicit acoustic or electric evoked clicks to control this aspect and verify neural synchrony.

Regarding the acquisition characteristics of the speech stimulus, its duration, intensity, and polarity are prominent concerns. The responses produced by short stimuli (<40 ms) are less susceptible to the cortical contribution, preserving the speech fundamental frequency representation and a shorter collection time^(27)^. Gabr and Hassaan^(10)^ performed differently from the other studies, as they used a long speech stimulus (206 ms).

The intensity of speech stimulus presentation is related to the peak latency and amplitude. The FFR peaks are visible from 10 dBSL^(28)^, which corresponds to the level in the studies, despite the different sound intensity measurement units they used. An increase in intensity usually decreases the wave appearance time and increases the tracing robustness; the opposite is also true^(28)^. This explains the latency and amplitude variations found in the studies.

The predominant use of alternating polarity without assessing the results with single polarity (rarefaction or condensation) can pose a problem. Alternating polarities and not replicating the stimulus with single polarity may enhance the CI artifact, as it favors the less frequent response components, including phase locking in the amplitude envelope^(5)^.

On the other hand, variations in electrode positioning do not cancel the CI electromagnetic artifacts or changes in the responses, as demonstrated in the cortical potentials with the electrodes positioned far from the CI^(29)^.

As in normal-hearing people, the stimulation rate can selectively affect the FFR result in CI users^(30)^. Onset response (waves V and A) latencies and amplitudes are the main components changed by faster presentation rates^(30)^. Thus, the increase in latencies and the irregularity in amplitudes of waves V and A can be caused by the different stimulation rates between studies.

The total number of sweepings aids in examination reproducibility. A minimum of 4,000 to 6,000 sweepings collected for verbal stimuli are expected, an interval that helps track subtle differences over time^(5)^. Mourad and collaborators^(11)^ collected lower values than recommended, which compromises the quality of their results.

The visualization window, in its turn, helps establish the response validity when it includes the pre-stimulation, stimulation, and post-stimulation time^(5)^. None of the studies included all this information, raising doubts about the validity of the responses. Also, criteria such as filters, impedance, and artifact rejection help maintain typical neural responses^(5)^, avoiding artifact contamination. However, they were ignored in most studies^(10,11,21,22)^ without justification.

Overall, the FFR acquisition parameters in CI users were not previously standardized in the investigations. Given the scarcity of comparable studies, the effect of the different protocols on the FFR results could not be assessed. Therefore, it is difficult to understand which variations directly affected the findings.

The analyses of the wave latencies and amplitudes reveal that the time domain furnishes data on changes in the neural transmission frequency of the transitory and sustained components of speech decoding in the auditory pathways^(5)^. Traditionally, latency and amplitude are used as indicators of neural delay and temporal synchronicity deficits in auditory processing disorder^(6-8)^. However, other measures can be employed to better understand the FFR recordings in CI users.

The analysis of the frequency domain, as performed in two studies^(11,22)^, exemplifies it. The mean square value (RMS) analyses, Fourier analysis, and stimulus-response correlation indicated that CI users may have weak decoding of the spectral characteristics involving the fundamental frequency and its harmonics. This agrees with findings in language and auditory processing disorders^(6-8)^, indicating a possible perceptual deficit.

Frequency domain analysis may be used as a differential analysis approach in the future to map the speech processing difficulties in CI users, as these measures are part of the person’s capacity to distinguish melodic and phonetic intonations^(5)^.

### Analysis of FFR results in CI users

The main concern regarding the results of the studies is the uncertainty of CI artifact removal combined with the high risk of bias. Given the duration of the speech stimuli used in the studies, the CI artifact may have overshadowed the neural response^(22,31)^. Hence, the FFR results may have been contaminated, as with the cortical auditory evoked potentials^(14)^.

For instance, the model response provided in the study^(10)^ is too similar to the speech stimulus to be true, and the morphology of the tracing has hints of the CI continuous current artifact. Also, the subjects’ age varied, which is expected to affect the cortical and FFR responses. Hence, the group with good cortical responses probably had greater FFR amplitudes, as the CI artifact may have caused sharper peaks in this study.

Despite the limited judgment in this type of visualization, evidence has proved that CI artifact is a random electrical peak that compromises the morphology and interrupts the occurrence and precision of the waves^(15)^. Therefore, even if the FFR results were present, they haven’t proved to be reliable.

Hoffman and Wouters^(31)^ provided a classic and detailed description of the procedure to remove from the recordings the CI artifact that is longer than the stimulus. Likewise, McLaughlin and collaborators^(14)^ and Presacco and collaborators^(15)^ demonstrated the procedure to attenuate the artifact in cortical responses with a clinically feasible single electrode approach. These alternatives were not considered in the studies included in this review^(10-13,21,22)^.

Moreover, the results of the studies^(10-13,21,22)^ did not consider the CI acoustic-electric processing time. The CI speech processor slows down and changes the naturality in acoustic-electric signal processing^(26)^. There is no evidence that this aspect was considered in the result analysis.

Contrary to the expected, one of the studies^(22)^ found more delayed FFR latencies in CI users than in normal-hearing people. The transduction process between the ears is eliminated in CI users due to the direct stimulation of the auditory nerve and, therefore, the central auditory pathways are activated at least 1.5 ms faster in CI users^(26)^. Hence, temporal compensation would be necessary for them to perceive the acoustic signals synchronously.

Various non-exclusionary reasons for speech perception difficulties in CI users were not controlled – e.g., low neural survival, partial insertion of the electrode set, quality of the signal furnished by the implant, current dispersion in the electrode bundle, and limited speech stimulation before deafness onset.

These reasons influence the sensory pattern the CI furnishes to the brain. The decoding of spectral and temporal details in the speech envelope tends to be impaired by the irregular activity of the remaining neural populations – which require the central auditory system to overcome distortions in the auditory patterns.

Nonetheless, the possibility that CI users have an impaired neural synchronization for speech decoding cannot be dismissed. The combination of the said factors may have interfered with the results and potentialized the delayed latencies, the smaller amplitudes, the higher VA complex values (slope, area, duration, and amplitude), and the low FFR spectral measures.

On the other hand, low wave amplitude values may indicate greater difficulty in speech perception, due to less neural activation in the auditory pathways over time. The higher VA complex measure values may reflect neural dyssynchrony in speech decoding. Also, the low spectral measures (HF, F0, and F1) may indicate deficits in the perception of prosodic and phonetic aspects of speech in CI users.

Since no approach is known to be valid to confirm the veracity of the FFR responses in CI users, further research must advance in this direction.

### Possible confounding factors related to FFR in CI users

Binkhamis and collaborators^(13)^ pointed out that the CI type (Cochlear Nucleus 22) and the bipolar speech processing strategy produce responses similar to the stimulus envelope with smaller amplitudes. They explained that this CI type is more susceptible to artifacts, and the bipolar stimulation produces smaller amplitudes because of the intracochlear reference electrode^(13)^.

Therefore, the CI artifact may have influenced these findings. However, since the CI type and processing strategy have been correlated with speech perception results^(32)^, these are considered confounding variables.

Complementarily, Gabr and Serag^(21)^ made evident that the age when the person begins using the CI is positively correlated with the VA complex measures. Rahman and collaborators^(12)^ observed that the mean time of CI use has a positive correlation with the latencies of waves V and E, which has a negative correlation with wave O. Altogether, these findings show the importance of an early cochlear implant to mature the auditory pathways, especially in the critical neural plasticity time.

The effect of these variables on FFR does not dismiss the interference of additional noncontrolled factors, as most studies reported only basic characteristics of the groups that were being compared. Since none of the studies used analysis methods to control the confounding factors (e.g., regression with propensity scores or covariables) and three studies^(10,11,22)^ did not homogenize the sample before the examination, a baseline imbalance is inferred.

Thus, the lack of control of confounding factors may have led to false results. The variables considered in the studies^(10-13,21,22)^ can be consulted to control their effects on the FFR results in future research.

### Limitations and clinical and research implications

The limitations of this study include the low number of studies addressing FFR in CI users, with few participants, without previously calculating the sample size, and with a high risk of bias combined with the use of systematized protocols.

Further studies with representative samples and more robust designs would enable meta-analyses and generalization of the results. Moreover, implementing more rigid statistical control techniques in relation to the possible confounding variables, acquisition, and analysis may minimize the measurement biases. Investing in research to validate approaches to minimize CI artifacts in FFR is particularly warranted, as they pose a challenge inherent to the examination.

## CONCLUSION

Although FFR can be applied to CI users, there is no consensus regarding an acquisition and analysis protocol to be used with this population. CI users have different FFR response patterns from those of normal-hearing people, according to the existing literature.

Given the methodological variations in the examination parameters and the lack of sample representativity, the findings of the studies cannot be generalized. Future studies should validate resources that can be incorporated or developed to improve the FFR acquisition and analysis techniques in CI users, including the minimization of CI artifacts, which is a prominent concern.
