# Supplementary material for: Frequency-Following Response (FFR) in cochlear implant users: a systematic review of acquisition parameters, analysis, and outcomes
Source: Codas. 2022 Jan 19;34(4):e20210116. doi: 10.1590/2317-1782/20212021116 (PMC9886122; doi:10.1590/2317-1782/20212021116)
Supplement: Translation

# Translation: Frequency-Following Response (FFR) em usuários de implante coclear: uma revisão sistemática dos parâmetros de aquisição, análise e resultados

**Authors:** Leonardo Gleygson Angelo Venâncio, Mariana de Carvalho Leal, Laís Cristine Delgado da Hora, Silvana Maria Sobral Griz, Lilian Ferreira Muniz

**Affiliations:** 1 Programa de Pós-graduação em Saúde da Comunicação Humana, Universidade Federal de Pernambuco – UFPE - Recife (PE), Brasil.; 2 Programa de Pós-graduação em Saúde da Comunicação Humana, Departamento de Cirurgia, Universidade Federal de Pernambuco – UFPE - Recife (PE), Brasil.; 3 Programa de Pós-graduação em Saúde da Comunicação Humana, Departamento de Fonoaudiologia, Universidade Federal de Pernambuco – UFPE - Recife (PE), Brasil.

**Keywords:** Implante coclear, Potenciais Evocados Auditivos do Tronco Encefálico, Fala, Transtornos da Percepção Auditiva, Eletrofisiologia, Revisão

## Abstract

**Objetivo:**

Caracterizar os parâmetros de aquisição, análise e resultados do exame Frequency Following Response (FFR) em usuários de implante coclear.

**Estratégia de pesquisa:**

As buscas foram realizadas nas bases Cochrane Library, Literatura Latino-Americana e do Caribe em Ciências da Saúde (LILACS), Ovid Technologies, PubMed, SciELO, ScienceDirect, Scopus, Web of Science e na literatura cinzenta.

**Critérios de seleção:**

Foram incluídos estudos sobre o FFR em usuários de implante coclear ou que os comparassem à indivíduos com audição normal, sem restrição de idade. Foram excluídos estudos secundários e experimentais. Não houve restrição de idioma e ano de publicação.

**Análise dos dados:**

Os dados foram analisados e redigidos de acordo com as etapas do Preferred Reporting Items for Systematic Reviews and Meta-Analyse (PRISMA) 2020. Para análise da qualidade metodológica foi utilizado o instrumento Joanna Briggs Institute Critical Appraisal Checklist for Analytical Cross Sectional Studies. As divergências foram resolvidas por um terceiro pesquisador.

**Resultados:**

Seis estudos atenderam aos critérios de inclusão. Apenas um estudo foi do tipo comparativo com grupo controle de indivíduos com audição normal. As variações nos parâmetros de aquisição foram comuns e as análises predominaram no domínio do tempo. Usuários de implante coclear apresentaram diferenças nos resultados do FFR quando comparados a indivíduos com audição normal, considerando a literatura existente. A maioria dos artigos teve baixa qualidade metodológica.

**Conclusão:**

Não existe padronização de um protocolo de aquisição e análise para o FFR em usuários de implante coclear. Os resultados são de alto risco de viés.

## INTRODUÇÃO

A evolução nas tecnologias do implante coclear (IC) permitiram a percepção da fala para a aquisição da oralidade. O IC é um dos recursos eficazes na reabilitação da perda auditiva (PA)^(1)^. Entretanto, variações no desempenho auditivo e linguístico dos usuários de IC têm sido frequentemente relatadas^(2)^, quando comparado aos indivíduos com audição normal. Isso sugere um possível processamento auditivo verbal distinto entre esses indivíduos.

Embora a origem exata desta variabilidade não seja totalmente definida, compreende-se que as características individuais, os fatores associados a PA e as características do IC influenciam na percepção da fala de usuários de IC^(3)^. Testes comportamentais são tradicionalmente utilizados para avaliar o processamento auditivo verbal, porém, como requerem a colaboração do indivíduo, sua aplicação tende a ser limitada em novos usuários de IC e crianças sem linguagem oral^(4)^.

Testes objetivos são, portanto, uma alternativa nesses casos. Dentre eles, o exame *Frequency Following Response* (FFR) é um procedimento não invasivo que mensura a codificação da fala pela atividade síncrona dos neurônios nas vias auditivas, especialmente no tronco encefálico, uma região importante para as funções de linguagem e audição^(5)^.

Evidências crescentes demonstram que o FFR auxilia no diagnóstico diferencial dos transtornos do processamento auditivo e da linguagem^(6-8)^. Para tanto, é necessário um registro adequado do exame, o qual depende da escolha dos parâmetros de aquisição e análise a serem utilizados^(9)^. Estudos com o FFR em usuários de IC utilizaram parâmetros similares aos utilizados em indivíduos com audição normal sem qualquer normatização prévia antes do teste^(10-12)^.

Esse cenário é desafiador, visto que a escolha inadequada de tais parâmetros favorece a contaminação dos registros do FFR pelo artefato elétrico gerado pelo IC que dificulta a interpretação clínica dos resultados obtidos, constituindo-se uma limitação inerente a sua possível aplicabilidade em usuários de IC^(13)^.

Várias técnicas para minimização de artefatos do IC foram descritas na literatura, entretanto, foram aplicadas aos potenciais corticais e/ou na resposta auditiva de estado estável^(14,15)^. A existência de um protocolo padronizado para obtenção de respostas livres de artefatos gerados pelo IC no FFR é desconhecida. Além disso, não está claro se existem diferenças válidas entre os padrões de respostas de usuários de IC e indivíduos com audição normal, dificultando identificar inconsistências e fragilidades nas evidências disponíveis para minimizá-las no futuro.

## OBJETIVO

Caracterizar os parâmetros de aquisição, análise e os resultados do exame FFR em usuários de implante coclear, por meio de revisão sistemática da literatura.

## ESTRATÉGIA DE PESQUISA

A Revisão sistemática da literatura foi redigida de acordo com os itens dos checklists *Preferred Reporting Items for Systematic Reviews and Meta-Analyses* (PRISMA) 2020^(16)^ e *The Meta-analysis of observational Studies in Epidemiology* (MOOSE)^(17)^. O protocolo completo da pesquisa está publicada sob registro CRD42020151073 no *International Prospective Register of systematic reviews* (PROSPERO).

O levantamento bibliográfico foi realizado nas bases Cochrane Library, Literatura Latino-Americana e do Caribe em Ciências da Saúde (LILACS), Ovid Technologies, Pubmed, SciELO, ScienceDirect, Scopus e Web of Science. A Literatura cinzenta foi consultada nas bases British Library Inside, DissOnline.de, OAIster, openDOAR, OpenGrey e The New York Academy of Medicine (NYAM). As buscas foram realizadas no dia 10 de março de 2020 e atualizadas no dia 10 de outubro de 2020.

A estratégia de busca incluiu descritores e palavras-chave que descrevem a população e o exame. Estes foram: “Cochlear Implants”; “Cochlear Implantation”; “Evoked Potentials, Auditory, Brain Stem”; “Implante Coclear”; “Potenciais Evocados Auditivos do Tronco Encefálico”; “Implantação Coclear”; “Implantes Cocleares”; “Respostas Evocadas Auditivas do Tronco Encefálico”; “Frequency-Following Response”; “Auditory brainstem response to complex sounds”; “envelope-following response”; “speech-evoked auditory brainstem response”; “Speech-evoked ABR”; subcortical steady-state response.

Nenhum limitador para o grupo comparador e o desfecho foi utilizado para garantir a recuperação do maior número possível de estudos relevantes. Os termos foram combinados e cruzados entre si utilizando os operadores booleanos (OR e AND) e quando possível, foram consideradas as variantes na ortografia, sinônimos, e sintaxe para maximizar a abrangência e garantir uma precisão razoável.

## CRITÉRIOS DE SELEÇÃO

Foram selecionados os estudos que examinaram usuários de IC e aqueles que os compararam com indivíduos com audição normal, desde que descreveram no mínimo um dos seguintes parâmetros de aquisição: transdutor, características do estímulo de fala (tipo, duração, intensidade e polaridade), orelha, montagem do eletrodo, taxa de apresentação do estímulo, reprodutibilidade, filtros, impedância e rejeição de artefatos. Não houve restrição quanto ao ano e idioma de publicação.

Foram excluídos estudos que incluíram sujeitos com síndromes neurológicas ou genéticas, malformações no tronco encefálico, distúrbios de linguagem ou audição e uso de fármacos com ação no sistema nervoso central. Também foram excluídos experimentos in vitro, ex vivo, com modelos animais, revisões, editoriais, índices, notícias, notas, cartas, abreviações, apêndices, relatórios, livros e capítulos de livros.

## ANÁLISE DOS DADOS

A análise dos dados ocorreu em quatro etapas (identificação, triagem, elegibilidade e inclusão)^(16)^ de forma independente pelos autores (L.G.A.V. e L.C.D.H.) e na ausência de concordância na etapa de inclusão, o estudo foi avaliado por um terceiro pesquisador (L.F.M) para tomada de decisão final.

Na etapa de identificação, foram selecionados os estudos apropriados por meio de consulta as bases de dados. O gerenciador de referências Mendeley Desktop (versão 1.19.8)^(18)^ foi utilizado para gerenciar, armazenar, compartilhar os estudos e para remover duplicatas. Na etapa de triagem, foi realizada a leitura do título e resumo para descartar estudos que não atendam aos critérios de seleção pré-estabelecidos e manter estudos possivelmente elegíveis.

Na etapa de elegibilidade, após a leitura do texto completo, foram decididos, quais estudos selecionados correspondiam à proposta da revisão: aquisição do FFR em usuários de IC ou a comparação entre usuários de IC e indivíduos com audição normal. No estágio de inclusão, foram agregados os estudos que atenderam todas as etapas anteriores, para a extração de dados.

Os dados coletados incluíram detalhes dos estudos (autor, ano e local), desenho do estudo, características amostrais (população, tamanho da amostra e idade), potenciais variáveis de confusão (variáveis sobre a PA e características do IC), protocolo de aquisição, análise e resultados do FFR. Esses foram agrupados em uma planilha do *software* Microsoft Office – Excel®.

A qualidade metodológica dos estudos foi analisada utilizando o instrumento *Joanna Briggs Institute (JBI) Critical Appraisal Checklist for Analytical Cross Sectional Studies* ^(19)^.

## RESULTADOS

### Seleção e características dos estudos

No total, foram identificados 6.639 artigos. Os artigos identificados através da busca sistemática nas bases de dados representaram 97,60% (N= 6.480) e os registros da literatura cinzenta corresponderam a 2,39% (N= 159). Foram triados 5.245 artigos após remoção das duplicidades. Na triagem pelo título e resumo, 5.238 artigos foram excluídos por não atenderem aos critérios de elegibilidade.

Após a triagem do título e resumo, sete artigos permaneceram para leitura do texto na íntegra. Na etapa de elegibilidade, excluiu-se um estudo^(20)^ por falta de dados para a orelha implantada, totalizando apenas seis estudos incluídos^(10-13,21,22)^ para extração de dados (Figura 1).

**Figura 1:** Fluxograma do processo de seleção dos estudos

As características dos seis estudos incluídos na revisão são apresentadas na Tabela 1. Os estudos foram produzidos entre os anos de 2015^(10)^, 2016^(11)^, 2017^(12)^, 2018^(21)^ e 2020^(22)^. Quatro estudos foram produzidos no Egito^(10-12,21)^, um no Reino Unido^(13)^ e outro no Irã^(22)^. Todos os estudos foram de delineamento observacional. Apenas um estudo^(22)^ teve como grupo comparador a população de crianças com audição normal. A distribuição da amostra variou por grupos subcategorizados que seguiram as questões originárias de cada pesquisa primária.

**Tabela 1:** Características dos estudos, método de análise, resultados e possível efeito das variáveis de categorização no FFR de usuários de IC

	Autor, ano e local										
	BinKhamis et al, 2019; United Kingdom^(13)^	Gabr et al, 2015; Egypt^(10)^		Gabr et al, 2018; Egypt^(21)^		Jarollahi et al, 2020; Iran (22)		Mourad et al, 2016; Egypt^(11)^	Rahman et al,2017; Egypt^(12)^		
Variáveis											
População e tamanho amostral (N)											
Grupo 1 (G1)	Usuários de implante coclear (12)	Bons registros corticais no potencial evocado auditivo cortical com estímulo de fala (9)		Quociente de melhoria da linguagem > 0.7 (20)		Usuários de implante coclear (20)		Usuários de implante coclear (10)	1 ano de uso do implante coclear (10)		
Grupo 2 (G2)	-	Pobres registros corticais no potencial evocado auditivo cortical com estímulo de fala (11)		Quociente de melhoria da linguagem < 0.7 (20)		Pessoas com audição normal (20)		-	2 anos de uso do implante coclear (12)		
Grupo 3 (G3)	-	-		-		-		-	3 anos de uso do implante coclear (9)		
Idade (anos)											
	39-60	2-6		4-10		8-10		5-1-	4-5		
Perda auditiva (n)											
Início da perda auditiva	Pós-lingual (12)	Pre- e pós-lingual (20)		Pré-lingual (40)		Pré-lingual (20)		Pré-lingual (10)	Pré-lingual (31)		
Grau da perda auditiva	NR	Severa a profunda (20)		Severa a profunda (40)		Profunda (20)		NR	NR		
Etiologia da perda auditiva	Caxumba (1), Idiopática (6), Meningite (2), Rubéola (2), trauma (1)	Hereditária (10), pós-febril (2), idiopática (8)		NR		NR		NR	Hereditária e idiopática (não especificada)		
Implante coclear (n)											
Orelha direita	6	9		NR		20		10	25		
Início de uso em anos	NR	2.2 para G1 e 3.2 para G2		4.1 para G1 e 3.8 para G2		4.1 para G1		NR	2.5 para G1, 2,1 para G2 e 1,5 para G3		
Tempo de uso médio em anos (grupo)	7.2	NR		2.6 para G1 e 2.4 para G2		4.6		NR	1.0 para G1, 2.0 para G2 e 3.0 para G3		
Marca (n)											
Abionic	-	5		NR		-			-		
Cochlear	6	5		-		20		-	-		
Med-El	6	10		-		-		10	22		
Neurelec	-	-		-		-		-	9		
Estratégia de processamento de fala											
ACE (MP1+2)	4	NE		NE		20		-	NE		
FS4	6	-		-		-		10	-		
SPEAK (BP+1/BP+2)	2	-		-		-		-	-		
**Método de análise do FFR**											
Pico da latência	Sim	Sim		Não		Sim		Sim	Sim		
Amplitude	Sim	Sim		Não		Sim		Sim	Sim		
Duração do complexo VA	Não	Não		Sim		Não		Não	Não		
Inclinação do complexo VA	Não	Não		Sim		Não		Não	Não		
Area do complexo VA	Não	Não		Sim		Não		Não	Não		
Amplitude do complexo VA	Não	Não		Sim		Não		Não	Não		
Magnitudes espectrais	Não	Não		Não		Sim		Não	Não		
Resultados do FFR por grupos											
	G1	G1	G2	G1	G2	G1	G2	G1	G1	G2	G3
Valores das latências (ms)											
V	8.22	9.60	10.3	-	-	11.9	9.51	2.59	9.35	8.65	7.27
A	12.1	12.9	14.7	-	-	13.4	10.9	4.5	10.6	9.86	9.20
C	NR	23.4	26.9	-	-	24.3	20.4	19.7	14.0	13.0	15.3
D	22.9	31.0	35.1	-	-	27.7	25.2	23.3	20.5	20.3	22.2
E	29.7	38.7	45.0	-	-	36.5	34.5	31.6	26.6	25.1	24.1
F	38.3	48.0	53.3	-	-	45.6	42.9	NR	38.0	39.0	39.8
O	46.2	56.9	63.0	-	-	54.1	51.1	NR	46.3	52.0	45.7
**Valores das amplitudes (µV)**											
V	NR	NR	NR	-	-	0.12	0.17	14.7	NR	NR	NR
A	0.96	NR	NR	-	-	-0.14	-0.20	9.74	NR	NR	NR
C	NR	NR	NR	-	-	-0.09	-0.14	2.11	NR	NR	NR
D	2.04	NR	NR	-	-	-0.26	-0.18	6.29	NR	NR	NR
E	0.79	NR	NR	-	-	-0.24	-0.17	7.27	NR	NR	NR
F	1.49	NR	NR	-	-	-0.18	-0.12	NR	NR	NR	NR
O	0.87	NR	NR	-	-	-0.12	-0.16	NR	NR	NR	NR
Valores de slope do complexo VA **(µV/ms)**	-	-	-	1.88	0.47	-	-	-	-	-	-
Area do complexo VA (µV X ms)	-	-	-	18.6	9.55	-	-	-	-	-	-
Amplitude do complexo VA (µV)	-	-	-	5.06	2.11	-	-	-	-	-	-
Duração do complexo VA (ms)	-	-	-	3.70	3.22	-	-	-	-	-	-
Valores das medidas de magnitude espectral (µV)											
HF	-	-	-	-		7.04	9.89	-	-	-	-
F0	-	-	-	-		2.25	4.58	-	-	-	-
F1	-	-	-	-		0.44	1.68	-	-	-	-
Efeito possível das variáveis de categorização no FFR	O modelo do implante coclear Nucleus 22 com a estratégia SPEAK resultou em respostas que se assemelhavam ao envelope de fala e que tinham menor amplitude antes da remoção de artefatos	NR		A idade de início do uso do implante coclear teve correlação positiva com as medidas de amplitude, área e slope do complexo VA		NR		NR	Tempo de uso médio do implante coclear em anos teve correlação positiva para as latências das ondas V e E e correlação negativa para a latência da onda O		

**Legenda:** ACE – *Advanced Combination Encoder*; BP – estimulação bipolar; F0 – frequência fundamental; F1 – primeiro formante; FS4 – *fine structure four*; HF – frequências altas; IC – implante coclear; LIQ *– language improvement quotient*; MP – estimulação monopolar; NE – Não especificado; NR – não reportado; RMS – valor eficaz; S-CAEPs – potencial evocado auditivo cortical com estímulo de fala; SPEAK, *Spectral Peak*

O tamanho amostral variou de 10^(11)^ a 40^(21)^ participantes usuários de IC e o intervalo de idade variou entre dois anos^(10)^ a 60 anos^(13)^. Cinco estudos foram conduzidos na população pediátrica^(10-12,21,22)^.

Todos os estudos foram classificados com alto risco de viés devido à baixa qualidade metodológica atribuível às limitações relacionadas aos critérios de inclusão, descrição dos participantes, bem como o local do estudo e identificação dos fatores de confusão (Tabela 2).

**Tabela 2:** Classificação da qualidade metodológica dos estudos seguindo os critérios do The Joanna Briggs Institute (JBI) Critical Appraisal Checklist for Analytical Cross Sectional Studies

	BinKhamis et al., 2019^(13)^	Gabr et al., 2015^(10)^	Gabr et al., 2018^(21)^	Jarollahi et al., 2020^(22)^	Mourad et al., 2016^(11)^	Rahman et al.,2017^(12)^
1. Os critérios para inclusão na amostra foram definidos claramente?	N	S	S	S	N	S
2. Os sujeitos e o local do estudo foram descritos detalhadamente?	N	N	N	S	N	N
3. A exposição foi medida de forma válida e confiável?	N	N	N	S	N	S
4. Foram utilizados critérios objetivos e padronizados para medir a condição?	N	S	N	S	N	S
5. Foram identificados os fatores de confusão?	N	N	N	N	N	N
6. Foram declaradas as estratégias para lidar com os fatores de confusão?	N	N	N	N	N	N
7. Os resultados foram medidos de maneira válida e confiável?	S	S	S	S	S	S
8. Foi usada análise estatística apropriada?	N	S	S	S	N	S
Adequado/Total	1/8	4/8	3/8	6/8	1/8	5/8

**Legenda:** S – Sim; N – Não; I – Indefinido; NA – Não se aplica

### Parâmetros de aquisição e análise do FFR em usuários de IC

A Tabela 3 apresenta os parâmetros de aquisição do FFR mais utilizados nos estudos em usuários de IC. Variações nos parâmetros foram frequentes, exceto, para o transdutor, tipo de estímulo, modo de estimulação e polaridade do estímulo. Todos os exames foram realizados com alto-falantes, estímulo de fala /da/ com polaridade alternada por meio da estimulação monoaural na orelha com o IC.

**Tabela 3:** Parâmetros de aquisição do FFR em usuários de IC dos estudos incluídos

**Estudo**	**Posicionamento do alto-falante**	**Características do estímulo de fala /da/**			**Orelha**	Montagem dos eletrodos*	**Taxa de apresentação (est/ms)**	**Número total de varreduras**	**Janela (ms)**	**Filtros (passa-alta – passa-baixa) (Hz)**	**Impedância dos eletrodos / rejeição de artefatos**	**Estratégia para diminuição do artefato do IC**
		Duração (ms)	Intensidade	Polaridade								
BinKhamis et al., 2019^(13)^	45° azimute, a 1,1 metro do microfone processador de fala do IC	40	70 dB-A	Alternada	OD/OE	Cz (+), A1 e A2 (-) e Fz (neutro)	9,1	10,000	NR	100 -3000	< 3 kΩ / NR	SIM
Gabr et al., 2015^(10)^	45° azimute, a 50 cm do lado da orelha com IC	206	70 dBHL	Alternada	OD/OE	Fz (+), M1 e M2 (-) e Fpz (neutro)	11,1	3,072	75	150– 1500	NR / NR	NR
Gabr et al., 2018^(21)^	45° azimute, a 1 metro do lado da orelha com IC	NR	70dBHL	Alternada	NR	Fz (+), M1 e M2 (-) e Fpz (neutro)	11,1	NR	75	150– 1500	NR / NR	NR
Jarollahi et al., 2020^(22)^	45° azimute, a 1 metro do nível da orelha com IC	40	50dB (SL)	Alternada	OD	Cz (+), A1 e A2 (-), Fpz (neutro)	9,1	4,000	60	100 -2000	< 5 kΩ / NR	NR
Mourad et al., 2016^(11)^	90° azimute, a 30 cm da cabeça	40	70 dBHL	Alternada	OD	Fpz, M1 e queixo (neutro)	2,1	1,000	60	30 -3000	<3kΩ / NR	NR
Rahman et al.,2017^(12)^	0° azimute, a 1 metro do participante	40	80 dBSPL	Alternada	OD/OE	Cz (+), M1 (-) e FPz (neutro)	10,9	6,000	62	100 – 2000	<5 kΩ / ±31 mV (miogênicos)	SIM

**Legenda:** (*) – baseado no sistema internacional 10-20 (A1 = lóbulo da orelha esquerda; A2 = lóbulo da orelha direita; Cz = vértex; Fpz = testa; Fz = fronte; M1 = mastoide esquerda; M2 = mastoide direita); (-) – eletrodo referência; (+) – eletrodo ativo; dB – decibel; est –estímulo; NR – não reportado; OD – orelha direita; OE – orelha esquerda; SPL – nível de pressão sonora

O posicionamento do alto-falante mais utilizado foi de 45° azimute em quatro estudos^(10,13,21,22)^, contudo, houve variação na distância do alto-falante em relação ao participante de 50cm^(10)^ a 1,1 metro^(13)^. Em relação as características do estímulo de fala, quatro estudos utilizaram o estímulo /da/ com 40 milissegundos (*ms*) de duração^(11-13,22)^ e três estudos apresentaram o estímulo de fala na intensidade de 70 dBHL^(10,11,21)^.

O FFR foi registrado utilizando eletrodos positivos, referência e terra fixados, respectivamente, nas regiões da parte superior da fronte (Fz), mastoides (M1 e M2) e testa (Fpz) em dois estudos^(10,21)^. Dois estudos utilizaram outra possibilidade de configuração^(13,22)^: vértex (Cz), lóbulos das orelhas (A1 e A2) e testa (Fpz). Os valores da taxa de apresentação do estímulo mais frequentes foram 9.1^(13,22)^ e 11.1^(10,21)^. O número total de varreduras variou entre 1.000^(11)^ a 10.000 estímulos^(13)^. A janela de visualização do exame mais frequente foi de 60 ms^(11,22)^ e 75 ms^(10,21)^.

Filtros passa-altas de 100Hz e passa-baixas de 2000 Hz foram selecionados em dois estudos^(12,22)^. A impedância dos eletrodos foi mantida abaixo de 3 quiloohms (kΩ)^(11,13)^ e <5 kΩ^(12,22)^ em dois estudos. Apenas um estudo^(12)^ adotou o critério de rejeição de artefato para minimizar interferências miogênicas. Dois estudos tentaram diminuir o artefato do IC com uma abordagem de aquisição com canal único^(13)^ e uso de jumper de eletrodo^(12)^.

Latência e amplitude de pico foram as medidas de análises predominantes nos seis estudos incluídos na revisão. Os estudos utilizaram os valores médios absolutos das latências e amplitudes das ondas V, A, C, D, E, F e O, bem como a duração, slope e área do complexo VA (Tabela 1).

Apenas um estudo^(22)^ utilizou a Análise de Fourier para examinar a representação do domínio da frequência por meio das medidas espectrais de frequência fundamental (F0), primeiro formante (F1) e altas frequências (HF). Dois estudos^(11,22)^, em contrapartida, analisaram o valor eficaz da amplitude (RMS) e a correlação cruzada entre o estímulo e as respostas do FFR.

### Resultados do FFR em usuários de IC

Os resultados do FFR apresentados pelos estudos são apresentados na Tabela 1. No geral, usuários de IC apresentaram atrasos nas latências, amplitudes reduzidas, maiores valores de slope, área, duração e amplitude do complexo VA e redução nas medidas espectrais (HF, F0 e F1) quando comparados aos dados estabelecidos pela literatura para indivíduos pediátricos^(8,23)^ e adultos^(7,24)^ com audição normal.

Devido a diversidade clínica e metodológica somada à disponibilidade limitada de amostra comparável e a existência de subgrupos categorizados da mesma população (usuários de IC) que atribui heterogeneidade aos achados, não foi possível realizar a meta-analise e a certeza da evidência, pois não houveram dados suficientes para calcular os tamanhos de efeitos.

Em síntese, esta revisão sistemática da literatura aborda uma perspectiva atual sobre a possível aplicação do FFR em usuários de IC. O FFR é um exame que tem se mostrado confiável para investigar a integridade do processamento da fala em diferentes populações^(6-8)^. Contudo, em usuários de IC a realização desse exame tende a ser limitada e prevê a interpretação dos resultados com várias ressalvas.

Para melhor compreensão, serão discutidos e analisados nas sessões seguintes os parâmetros de aquisição e análise do FFR em usuários de IC, bem como os resultados encontrados e os efeitos possíveis das variáveis de confusão relacionadas ao exame.

### Análise dos parâmetros de aquisição e análise utilizados no FFR de usuários de IC

Variações nos parâmetros de aquisição do FFR afetam os registros do exame^(5,25)^. Como todos os estudos apresentaram os estímulos por meio de alto-falantes e não mencionaram calibração prévia, esperava-se a compensação temporal devido à distância entre o transdutor e o IC e o período que o processador do IC requer para processar os sinais acústicos^(26)^. Além disso, houve falha por parte dos autores em não eliciar um clique evocado (acústico ou elétrico) para controlar esse aspecto e verificar a sincronia neural.

Em relação as características de aquisição do estímulo de fala, a duração, intensidade e polaridade do estímulo são uma preocupação proeminente. Estímulos curtos (<40*ms*) produzem respostas menos susceptíveis a contribuição cortical, preservando a representação da frequência fundamental da fala e menor tempo de coleta^(27)^. Gabr e Hassaan^(10)^ por utilizar um estímulo de fala de longa duração (206 ms), contrariaram os demais estudos.

A intensidade de apresentação do estímulo de fala está relacionada a latência e a amplitude dos picos. Os picos do FFR são visíveis a partir de 10 dB SL^(28)^, nível correspondente aos estudos, apesar das diferentes unidades de medidas de intensidade sonora adotadas. O aumento dessa intensidade provoca, geralmente, a diminuição no tempo de aparecimento das ondas e aumenta a robustez dos traçados e vice-versa^(28)^. Esse fato explica as variações nas latências e amplitudes encontradas nos estudos.

O predomínio do uso da polaridade alternada sem a avaliação dos resultados com polaridade única (rarefação ou condensação) pode ser considerado um problema. Pois, a alternância das polaridades sem a replicação com estímulos de polaridade única pode levar a acentuação do artefato gerado pelo IC ao favorecer os componentes de menor frequência da resposta, incluindo o *phase locking* no envelope de amplitude^(5)^.

Por outro lado, variações nos posicionamentos dos eletrodos não anulam os artefatos eletromagnéticos gerados pelo IC ou modificações nas respostas, como demonstrado nos potenciais corticais com os eletrodos posicionados distantes do IC^(29)^.

A taxa de estimulação pode afetar seletivamente o resultado do FFR em usuários de IC, assim como acontece em indivíduos com audição normal^(30)^. Latências e amplitudes das respostas *onset* (onda V e A) são os principais componentes que sofrem modificações com taxas de apresentação mais rápidas^(30)^. Dessa forma, o aumento nas latências e a irregularidade nas amplitudes das ondas V e A podem ter sido provocadas pelas diferentes taxas de estimulação entre os estudos.

O número total de varreduras auxilia na reprodutibilidade do exame. Espera-se um mínimo de 4000 a 6000 varreduras coletadas para os estímulos verbais, intervalo que favorece rastrear diferenças sutis ao longo do tempo^(5)^. Mourad e colaboradores^(11)^ coletaram valores inferiores aos recomendáveis, o que compromete a qualidade dos seus resultados.

A janela de visualização, por sua vez, auxilia na determinação da validade da resposta quando inclui o período pré-estímulo, momento de estimulação e pós-estímulo^(5)^. Nenhum estudo incluiu todas essas informações, gerando dúvidas quanto a validade das respostas. Além disso, critérios como filtros, impedância e rejeição de artefatos servem para conservar a resposta neural típica^(5)^, evitando a contaminação por artefatos. Eles foram ignorados na maioria de estudos^(10,11,21,22)^, não sendo justificado seu desuso.

Em conjunto, os parâmetros de aquisição do FFR em usuários com IC não foram padronizados previamente nas investigações. Devido à escassez de estudos comparáveis, não foi possível avaliar o efeito dos diferentes protocolos nos resultados do FFR. Portanto, torna-se difícil compreender quais variações afetariam diretamente os achados.

As análises das latências e amplitudes das ondas revelam que o domínio do tempo fornece dados sobre as mudanças na periodicidade da transmissão neural dos componentes transitórios e sustentados de codificação da fala nas vias auditivas^(5)^. Tradicionalmente, latência e amplitude são utilizadas como indicadores de atraso neural e déficits no sincronismo temporal no transtorno do processamento auditivo^(6-8)^. Porém, outras medidas podem ser empregadas para maior entendimento dos registros do FFR em usuários de IC.

A análise no domínio da frequência é um exemplo disso, como conduzida por dois estudos^(11,22)^. As análises do valor quadrático médio (RMS), análise de Fourier e correlação entre estímulo-resposta indicaram que indivíduos com IC podem possuir uma fraca codificação das características espectrais envolvendo a frequência fundamental e seus harmônicos, concordando com achados no transtorno do processamento auditivo e de linguagem^(6-8)^, indicando possível déficit perceptual.

Analisar o domínio da frequência pode futuramente ser uma abordagem analítica diferencial para mapear as dificuldades do processamento de fala de usuários de IC, já que essas medidas participam na capacidade do indivíduo realizar distinções de entonações melódicas e fonéticas^(5)^.

### Análise dos resultados do FFR em usuários de IC

A principal preocupação em relação aos resultados dos estudos é a incerteza da remoção dos artefatos gerados pelo IC associado ao alto risco de viés. Devido a duração dos estímulos de fala utilizados nos estudos, o artefato suscitado pelo IC se sobreporá à resposta neural^(22,31)^. Dessa forma, os resultados do FFR podem ter sido contaminados de modo semelhante como ocorre com os potenciais evocados auditivos corticais^(14)^.

Por exemplo, a resposta modelo fornecida no estudo^(10)^ parece demasiadamente com o estímulo de fala utilizado para ser verídico e a morfologia do traçado apresenta indícios do artefato de corrente contínua gerado pelo IC. Além disso, a idade variou entre os indivíduos e por isso esperava-se que esse fator incidisse sobre as respostas corticais e do FFR. Então, é provável que o grupo com boas respostas corticais tenham apresentado maiores amplitudes no FFR devido ao artefato do IC que pode ter provocado picos mais pronunciados nesse estudo.

Embora o julgamento nesse tipo de visualização seja limitado, evidências provaram que o artefato suscitado pelo IC é um pico elétrico aleatório que compromete a morfologia e interrompe a ocorrência e precisão das ondas^(15)^. Portanto, mesmo que os resultados do FFR tenham sido considerados presentes, não há indicativos que sejam confiáveis.

Hoffman e Wouters^(31)^ forneceram uma descrição clássica e detalhada do procedimento de remoção do artefato de IC para gravações que se sobrepõem no tempo à duração do estímulo. De maneira semelhante, McLaughlin e colaboradores^(14)^ e Presacco e colaboradores^(15)^ demonstraram o procedimento para atenuar o artefato de respostas corticais usando uma abordagem de eletrodo único clinicamente viável. Essas alternativas não foram levantadas pelos estudos incluídos nesta revisão^(10-13,21,22)^.

Além disso, os resultados dos estudos^(10-13,21,22)^ não consideraram o tempo de processamento acústico-elétrico do IC. O processador de fala do IC lentifica e modifica a naturalidade do processamento dos sinais acústicos-elétrico^(26)^. Não há evidência de que esse aspecto tenha sido considerado na análise dos resultados.

Em contraposição ao que se esperava, um dos estudos^(22)^ descobriu latências no FFR mais atrasadas para os usuários de IC do que em indivíduos com audição normal. O processo de transdução entre as orelhas é eliminado em usuários de IC devido a estimulação direta do nervo auditivo. Por essa razão, a ativação das vias auditivas centrais é pelo menos 1,5ms mais rápida em indivíduos com IC^(26)^. Assim, para que os sinais acústicos fossem percebidos de forma síncrona, seria necessário a compensação temporal.

Diversas razões não excludentes de dificuldade de percepção da fala em usuários de IC não foram controladas, tais como, baixa sobrevivência neural, inserção parcial do conjunto de eletrodos, qualidade do sinal fornecido pelo implante, dispersão de corrente no feixe de eletrodos e estimulação limitada com a fala antes do início da surdez.

Essas razões influenciam o padrão sensorial fornecido ao cérebro pelo IC, pois a codificação dos detalhes espectrais e temporais do envelope de fala tende a ser prejudicada pela atividade irregular das populações neurais remanescentes que demandam que o sistema auditivo central supere distorção nos padrões auditivos.

Embora não se descarte a possibilidade que usuários de IC tenham um comprometimento na sincronização neural para codificação da fala. A combinação dos fatores mencionados pode ter interferido nos resultados e potencializado os atrasos nas latências, a redução das amplitudes, os maiores valores (slope, área, duração e amplitude) do complexo VA e a redução nas medidas espectrais do FFR.

Por outro lado, a redução nos valores das amplitudes das ondas podem indicar uma maior dificuldade de perceber a fala pela menor ativação neural nas vias auditivas ao logo do tempo. Os maiores valores das medidas do complexo VA podem refletir uma dissincronia neural na codificação da fala. E a redução nas medidas espectrais (HF, F0 e F1) podem indicar déficits na percepção dos aspectos prosódicos e fonéticos da fala em usuários de IC.

Como não é conhecida uma abordagem válida para confirmar a veracidade das respostas obtidas no FFR de usuários de IC, pesquisas futuras devem avançar nesse sentido.

### Possíveis fatores de confusão relacionadas ao FFR em usuários de IC

Binkhamis e colaboradores^(13)^ apontaram que o tipo de IC (Cochlear Nucleus 22) e a estratégia de processamento de fala bipolar produzem repostas que se assemelham ao envelope do estímulo com amplitudes reduzidas. Eles justificaram que esse tipo de IC é mais susceptível a artefatos e a estimulação bipolar produz amplitudes reduzidas devido ao eletrodo de referência ser intra-coclear^(13)^.

Então, é possível que o artefato gerado pelo IC tenha influenciado nesses achados. Porém, como o tipo de IC e a estratégia de processamento do IC têm sido correlacionado aos resultados de percepção de fala^(32)^, consideram-se essas variáveis como confundidoras.

De maneira complementar, Gabr e Serag^(21)^ evidenciaram que a idade de início do uso do IC é correlacionada positivamente com as medidas do complexo VA. Rahman e colaboradores^(12)^ observaram que o tempo de uso médio do IC possui uma correção positiva com as latências das ondas V, bem como da onda E que sofre correlação negativa com a onda O. Em conjunto, esses achados mostram a importância da implantação coclear precoce para o amadurecimento das vias auditivas, especialmente durante o período crítico de plasticidade neural.

O efeito dessas variáveis no FFR não descarta a interferência de fatores adicionais não controlados, pois a maioria dos estudos relataram apenas características básicas dos grupos que estavam sendo comparados. Como nenhum estudo utilizou métodos de análise para controlar os fatores de confusão (por exemplo, regressão com escores de propensão ou covariáveis) e três estudos^(10,11,22)^ não homogeneizaram a amostra antes da realização do exame, infere-se um desequilíbrio na linha de base.

Portanto, a falta de controle sobre os fatores de confusão pode ter propiciado resultados falsos. As variáveis que foram consideradas nos estudos^(10-13,21,22)^ podem ser consultadas com o objetivo de controlar seus efeitos nos resultados do FFR em trabalhos futuros.

### Limitações e Implicações clínicas e para pesquisa

As limitações desta revisão incluem: o baixo número de estudos sobre o FFR em usuários de IC com um número limitado de participantes, os quais não passaram por cálculo prévio do tamanho da amostra, e o alto risco de viés associado ao uso de protocolos sistematizados.

Novos estudos com amostra representativa e com delineamentos mais robustos facilitarão a realização de metanálises e a generalização dos resultados. Além disso, a implementação de técnicas de controle estatístico mais rígidas em relação às possíveis variáveis de confusão, aquisição e análise do exame podem minimizar os vieses de aferição. Em particular, investir em pesquisas que validem abordagens para a minimização de artefatos gerados pelo IC no FFR, pois constitui-se um desafio inerente ao exame.

## CONCLUSÃO

Apesar de ser possível a aplicação do FFR em usuários de IC, não existe consenso quanto a um protocolo de aquisição e análise a ser utilizado nessa população. Usuários de IC apresentam diferenças nos padrões de resposta no FFR quando comparados a indivíduos com audição normal, considerando a literatura existente.

Devido às variações metodológicas nos parâmetros do exame e à falta de representatividade amostral, não é possível generalizar os achados dos estudos. Sugere-se que estudos futuros validem os recursos que podem ser incorporados ou desenvolvidos para melhorar as técnicas de aquisição e análise do FFR em usuários de IC, incluindo a minimização de artefatos gerados pelo dispositivo auditivo que é uma preocupação proeminente.
